# Perceptions of causal attribution and attitudes to genetic testing among people with schizophrenia and their first-degree relatives

**DOI:** 10.1038/s41431-022-01116-8

**Published:** 2022-05-16

**Authors:** Melissa B. R. Cullen, Bettina Meiser, Kristine Barlow-Stewart, Melissa Green, Paul S. Appelbaum, Vaughan J. Carr, Murray J. Cairns, M. S. Lebowitz, Rajneesh Kaur

**Affiliations:** 1 School of Medicine, University of New South Wales, Sydney, Australia; 2 Prince of Wales Clinical School, University of New South Wales, Sydney, Australia; 3 Sydney Medical School, University of Sydney, Sydney, Australia; 4 School of Psychiatry, University of New South Wales, Sydney, NSW 2052 Australia; 5 Neuroscience Research Australia, Sydney, NSW 2031 Australia; 6 Department of Psychiatry, Columbia University Vagelos College of Physicians & Surgeons, New York, USA; 7 Center for Research on Ethical, Legal & Social Implications of Psychiatric, Neurologic & Behavioral Genetics, Columbia University Irving Medical Center, New York, USA; 8 School of Biomedical Sciences and Pharmacy, University of Newcastle, Newcastle, Australia; 9 Hunter Medical Research Institute, Newcastle, NSW 2305 Australia

**Keywords:** Human behaviour, Genetic testing

## Abstract

Rapid advances in the genetics of psychiatric disorders mean that diagnostic and predictive genetic testing for schizophrenia risk may one day be a reality. This study examined how causal attributions for schizophrenia contribute to interest in a hypothetical genetic test. People with schizophrenia and first-degree relatives of people with schizophrenia were recruited through a schizophrenia research bank and mental health organisation. Semi-structured telephone interviews were conducted with 13 individuals with schizophrenia and 8 first-degree relatives. Transcripts were subjected to a qualitative analysis using the thematic analysis framework. Five themes were developed: (i) “It is like a cocktail”, with most participants aware that both genetic and environmental factors contributed to causation, and many mentioning the positive impact of genetic causal explanations; (ii) “Knowledge is power” (i.e., in favour of genetic testing); (iii) Genetic testing provides opportunities for early intervention and avoiding triggers, with participants citing a wide range of perceived benefits of genetic testing but few risks; (iv) Views on reproductive genetic testing for schizophrenia risk with a few participants viewing it as “playing God” but not necessarily being against it; and (v) “It snowballs”, whereby participants’ understanding of genetics was sophisticated with most believing that multiple rather than single genes contributed to schizophrenia. In conclusion, many individuals had a sound understanding of the role of genetic testing if it were to become available, with evidence of insight into the role of multiple genes and the contribution of other risk factors that may interact with any inherited genetic risk.

## Introduction

Schizophrenia is a debilitating psychiatric condition characterized by delusions, hallucinations, disorganized thinking and speech, impaired cognitive function and a blunted affect [1]. It has a lifetime prevalence of 0.3–0.7% and contributes significantly to global burden of disease [2]. Family, twin and adoption studies consistently indicate that there is a strong genetic contribution to the aetiology of schizophrenia, with heritability estimates of approximately 80% [3].

Advances in molecular genetics over the past decade have led to clinically applicable tests for rare variants leading to schizophrenia, and comparative genomic hybridization arrays and other technics aiming at detecting copy number variants are being implemented for schizophrenia in a growing number of countries [4–6]. However, genome-wide association studies (GWAS) have revealed the genetic component of schizophrenia to be complex, heterogenous and largely polygenic [7]. In particular, a single analysis of all available schizophrenia GWAS samples identified 145 loci, which met genome-wide significance. While this study and others have implicated several genetic regions in the pathophysiology of schizophrenia, the key finding has been that a significant component of the polygenic nature of schizophrenia can be attributed to thousands of *common* alleles of *small* effect that individually do not attain significance [8].

This rapidly advancing understanding of genetics opens the door to a new era of clinical technology and personalized medicine. Polygenic risk scores (PRSs), i.e., genetic risk estimates based on common variation in multiple genetic loci, show promise in aiding clinical decision-making in psychiatry [9]. The opportunity exists to optimise existing genetic risk estimates for schizophrenia, which are currently presented as ranges based on epidemiological data, to provide personalised risk information [10]. Polygenic risk scores show some promise in schizophrenia. They are able to discriminate between groups of unrelated schizophrenia cases and controls with reasonable sensitivity and specificity, and they could be used in people with symptoms for risk stratification [11]. However, they are not yet sufficiently accurate for clinical diagnosis or prediction.

It also should be noted that there is considerable overlap among common single nucleotide polymorphisms (SNPs) for five psychiatric disorders, and hence that PRS scores are not specific to schizophrenia [7]. Therefore, while they may be able to distinguish people with schizophrenia from healthy people, they do not necessarily distinguish schizophrenia from other disorders.

Future refinement of polygenic risk scores (PRS) for psychiatric disorders may create opportunities for prevention, such as through lifestyle modification, and allow for an individualised approach to management [12]. Genetic testing may enable patients and families to make informed reproductive decisions through prenatal or pre-implantation testing [13]. Additionally, a genetic explanation for one’s condition may provide psychological benefits, including a reduction in self-blame and reassurance that personal life choices are not a major reason for illness [12]. Despite the high likelihood that genetic risk information for psychiatric disorders would be probabilistic rather than conveying diagnostic certainty, learning of a genetic test result may also cause psychological distress or affect perceptions of treatability [14]. Adequate education and counselling is essential to ensure perceived benefits are balanced with potential risks [15].

Patient and family interest in genetic counselling for schizophrenia risk is well-established [16–18]. Those who have received genetic counselling for schizophrenia risk report high rates of satisfaction and a reduction in psychological distress, stigma, and self-blame, and endorse its helpfulness and necessity, even without receiving a personalised risk result [19, 20]. Overestimation of the risk of recurrence is, however, common in family members, and this is associated with reproductive decisions favouring fewer or no children, highlighting the importance of genetic counselling to facilitate accurate genetic risk perceptions [21].

There is ample evidence from quantitative studies to suggest a strong level of interest in genetic testing from both people with schizophrenia and their genetic relatives. However, how this population makes sense of genetic risk information has not been examined in-depth, and factors that contribute to decision-making about determining genetic risk remain understudied. This study therefore aims to explore perceived causal attributions for schizophrenia, including the degree to which a genetic model is endorsed, and the impact of these attributions on the perceived stigma of schizophrenia. This study also aims to explore attitudes towards diagnostic and predictive genetic testing, using different risk frames, and assess interest in reproductive genetic testing. The findings will inform educational needs in relation to the provision of information about familial risk to patients and may also guide genetic counselling practice.

## Materials and methods

### Participants

To be eligible, participants either had to: (i) have a diagnosis of schizophrenia or schizoaffective disorder (affected individuals) or (ii) be a first-degree relative of an individual with a diagnosis of schizophrenia or schizoaffective disorder (unaffected individuals). First-degree relatives must not have had a diagnosis of schizophrenia or schizoaffective disorder themselves. For both affected and unaffected individuals, additional eligibility criteria were: aged 18 years or older and being able to speak English proficiently.

### Recruitment

Purposive sampling [22] of people with schizophrenia and first-degree relatives of people with schizophrenia was utilised for this study, because this population has an important perspective on the topic being investigated. Participants for this study were recruited through two pathways: (i) the Australian Schizophrenia Research Bank (ASRB) participant register, and (ii) One Door Mental Health, an Australian organization that designs and delivers expert mental health programs for people with mental illness, including schizophrenia [23]. The ASRB was established in 2007 and aims to facilitate research through collection of clinical and biological information from over 1000 individuals with schizophrenia, drawn from five Australian states and territories [24]. The ASRB clinical participants have a confirmed diagnosis of schizophrenia or schizoaffective disorder, according to Diagnostic and Statistical Manual of Mental Disorders, fourth edition (DSM-IV) / International Classification of Diseases, Tenth Revision (ICD-10) diagnostic criteria. All participants had the option of registering to be contacted about participation in future schizophrenia-focused research projects. Participants in the cohort of biological relatives were recruited through resources associated with ASRB collaborators, including media advertisements, inpatient, outpatient and community mental health service providers, non-government organizations, and rehabilitation services.

### Procedure

Recruitment of ASRB participants was facilitated by invitations sent by mail to eligible individuals (who had consented to be recontacted by the ASRB for further studies), to obtain explicit consent to be contacted by the research team for potential inclusion in this study. Only those ASRB participants who consented for their contact details to be forwarded to the research team were contacted for potential participation in this study; consenting ASRB participants were sent a Participant Information Sheet and Consent Form along with an invitation letter. This was followed with a phone call to ascertain interest in participating, answer any questions, and arrange a time for an interview. Individuals were asked to return the signed consent form prior to the interview. During the phone call, individuals were asked whether they had unaffected first-degree relatives who might be interested in participating in the study; if so, they were asked for their relatives’ contact details. Unaffected first-degree relatives were recruited in the same manner as the ASRB participants (i.e., first by letter to obtain consent for further contact by the research team). To recruit One Door Mental Health participants, a study invitation was published on the One Door Mental Health website [23] Interested individuals were asked to contact the study co-ordinator, and the ensuing interview procedures were analogous to those for participants recruited through the ASRB. Participants recruited through both pathways were provided with a $50 grocery voucher to compensate for their time.

Semi-structured telephone interviews were conducted by MC and RK, using a guide outlining the major topics to be covered during the interview, whilst leaving wording and sequencing of questions open (Supplementary File 1). MC is a medical student trained in qualitative methodology and RK is an experienced qualitative researcher with a background in public health. Interviews explored causal attributions for schizophrenia, interest in genetic testing for schizophrenia, extent to which the degree of certainty of test result impacts on interest in genetic testing, perceived benefits and risks of genetic testing, and attitudes to reproductive genetic testing. In early interviews emergent views were used to guide certain lines of questioning in subsequent interviews, to capture divergent points of view [25]. When data saturation was achieved, sampling was discontinued. Interviews were digitally recorded and transcribed by a professional transcription company. Transcripts were deidentified and participants were assigned pseudonyms. To preserve anonymity, participants’ ages have been omitted from the quotes in the results section.

Peer review and member checking were strategies used to ensure rigor and improve credibility of the findings. Peer review was performed through discussions during researchers’ meetings. Member checking was conducted towards the end of each of interview by summarizing the main points/themes emerging from the interview and confirming these with the participant. As the interviewers, MC and RK acknowledged inherent biases and participated in ongoing reflection.

### Data analysis

The data analysis was guided conceptually by thematic analysis [25], which is based on an essentialist/realist approach (i.e., the understanding that participants express through language their experiences of reality, resulting in identification of themes at a semantic/explicit level) [26]. Interview transcripts were analysed by MC, RK, BM, and KBS [25]. BM and KBS are experienced qualitative researchers, and experts in psychosocial aspects of genetics and genetics education, respectively. Initial themes were identified by MC and discussed with BM to confirm the reliability of the coding scheme and further refine and expand on emergent themes. RK and KBS reviewed the codes, and any discrepancies were resolved by discussion to arrive at an agreed upon set of themes and subthemes. The remaining transcripts were then coded by MC and BM using the qualitative data analysis software NVivo Version 12.0 to help with organisational aspects of data management.

## Results

Interviews were conducted between June 2018 and May 2020. Of 50 participants invited by the ASRB, 13 affected individuals consented to being contacted by the research team; one did not meet eligibility criteria, and 12 were interviewed (24% participation rate amongst ASRB participants). An additional three unaffected relatives of ASRB participants were interviewed. One affected and five unaffected participants were recruited through One Door Mental Health. A total of 21 interviews (13 with affected participants and 8 with unaffected relatives) were transcribed and analysed. Table 1 shows participants’ sociodemographic, clinical status, and recruitment source characteristics separately for affected and unaffected participants. The mean age of participants was 55 years (SD = 14.1). Nine were male (5 affected, 4 unaffected) and 12 female (8 affected and 4 unaffected). Ten of the affected participants had never been married, compared to only 3 of the unaffected relatives. Interviews ranged from 13 to 90 min in duration, with a mean interview time of 31 (SD = 20.9) minutes.

**Table 1:** Sociodemographic characteristics of participants (*n* = 21).

	Affected participants	Unaffected relatives	Total (*n* = 21)
	(*n* = 13)	(*n* = 8)	
**Age**			
Mean (SD) (years)	53.5 (11.7)	56.3 (18.2)	54.6
Range (years)	36–80	25–77	25–80
	*n*	*n*	*n*
**Gender**			
Female	8	4	12
Male	5	4	9
**Country of birth**			
Australia	11	7	18
Other	2	1	3
**Level of education**			
Year 10	0	1	1
Year 12	2	2	4
Trade or apprenticeship	1	0	1
TAFE/college certificate/diploma	5	3	8
Bachelor’s degree	5	2	7
**Marital status**			
Never married	10	3	13
Married or de facto	1	4	5
Widowed	1	1	2
Divorced	1	0	1
**Recruitment source**			
Australian Schizophrenia Research Bank	12	3	15
One Door Mental Health	1	5	6

Thematic analysis identified 5 themes.

### “It is like a cocktail” - Causal attributions for schizophrenia

Quotes to illustrate causal attributions for schizophrenia are shown in Table 2, including the number of participants who mentioned particular aspects of this theme. The majority of participants felt that schizophrenia arose as a result of a gene/environment interaction, as exemplified by one participant, who thought “it is like a cocktail with all the elements of a fire and then it suddenly ignites.” By contrast, only two participants considered schizophrenia as being caused almost completely by genetic factors. Several thought it was caused almost completely by environmental factors, and all but one of these had a personal diagnosis of schizophrenia. Participants mentioned several specific environmental factors, which they believed “contributed to” or “triggered” their illness or that of their relative. More than half mentioned the use of illicit drugs, in particular marijuana. Stress and having had a difficult childhood, including child sexual abuse, were also mentioned as contributing factors.

**Table 2:** “It is like a cocktail” - Causal attributions for schizophrenia.

Themes	Quotations
Role of genes versus environment – it is like a cocktail	*Acknowledges gene environment interaction (12)*
	Yes, I think it is like a cocktail, like all the elements of a fire and then it suddenly ignites. (Oliver, unaffected)
	If you have got a family genetic factor, you are much more likely to get it just if you are under a lot of stress, because I have known people in my life who have been under a lot of stress and they never get mentally ill. Other people do, so to me, it needs both. It needs stresses and a genetic factor. (Justine, affected)
	I would say it is a biochemical disorder, which has a lot of factors which cause it and contribute to it, but basically there is a predisposition and this is how that person deals with it. (Robyn, affected)
	*Considers schizophrenia caused almost completely by genetic factors (2)*
	Like genetic? Yes yeah. A lot, because I think things like schizophrenia do run in families. (Justine, affected)
	Well as far as I can find out it is probably maybe about 90% or even more is inherited. (Michael, unaffected)
	*Considers schizophrenia caused almost completely by environmental factors (5, 4 affected)*
	It was abuse and then he took drugs, he took LSD...All this contributed to his illness. I think it is environmental. (Andrea, unaffected)
	Yes, well I have not had anybody else in the family, and so I am not quite sure about hereditary. (Susan, affected)
	I do not think much of the genetic connection. I see it as environmental. (Elisabeth, affected)
	Yeah, I think nuture is a massive part. (Will, affected)
Considers specific environmental factors and experiences	*Use of illicit drugs (11)*
	Smoking marijuana was the thing that triggered it, sent me over the edge. (Ian, affected)
	But I do get angry when I think that maybe if the drugs have not been involved, it may not have come to the fore as early as it did with [daughter]. (Annabel, unaffected)
	I have heard anecdotal stuff from other people in the mental health community that it was caused by drug use. (Patricia, affected)
	*Stress* (7)
	And that-I got a lot of stress from that and I believe that triggered it, but you know, it is-but I also believe there is an underlying genetic predisposition to it. (Harry, affected)
	Ah stress would be a big one, yes stress and drugs I think of the big two ones. (Vince, affected)
	*Difficult childhood (5)*
	In my brother’s case, he had a very troubled childhood …He abused him sexually when he was a child. (Andrea, unaffected)
	I know my upbringing was not very good so I think that it probably caused some of my issues. (Eleanor, affected)
Positive impact of a genetic explanation	*Knowledge of family history would have been helped (3)*
	No, no, everyone kept everything hush-hush and did not talk about it at all. It was only after I got diagnosed that they said the two uncles had schizophrenia as well… I thought if I could, you know, if I knew that before, I would have probably been more aware of the signs and been able to seek help earlier think. (Vince, affected)
	People just cover it up, but had I known that my cousin … got schizophrenia, I seriously think it would have helped me a lot (Will, affected)
	*Reduces responsibility for condition and helps with acceptance (2)*
	I think it would be very helpful, then a person will know what actually caused it. (Andrea, unaffected)
	*Reduces stigma (3)*
	If people saw it more as an illness, sickness, then it would reduce the stigma, and the genetic explanation does explain it is an illness so I think it would reduce the stigma an awful lot. (Robyn affected)
	*Reduces self-blame (3)*
	Oh! I think it would make it better. I’d say if they said it was 50% genetic or 100% genetic, I think, you know, there’d be no self-blame, it’s just one of those things that happen genetically.’ (Vince, affected)
	It probably helps with acceptance, because you think…it’s not my fault, can’t do anything about it, so I have not done much wrong (Harry, affected)
Negative impact of a genetic explanation	*A genetic explanation would lead to blaming of parents*
	I think the people with schizophrenia if they said it was genetic, it would add fuel to the fire against their parents, you know, you gave me these rotten genetics. (Robyn, affected)

When asked about the hypothetical impact of having a genetic causal explanation for schizophrenia, many participants suggested that such explanations would have a positive impact. A few participants, who were unaware of any family history before they were diagnosed, mentioned that it would have been helpful to know about their family history, including that they would have been able to seek help earlier. Two affected individuals mentioned that a genetic explanation would be helpful and considered that it might contribute to acceptance of the condition. A genetic explanation was also seen as a means of reducing stigma by several participants in that it legitimised schizophrenia as a medical condition, thus shifting blame from the affected person and reducing self-blame. Conversely, one participant believed a genetic explanation would have little impact on the way people viewed someone with schizophrenia, explaining that people make judgments based on their experiences regardless of perceived cause. Only one participant believed that having a genetic explanation would have a negative impact, in that a genetic explanation may shift blame towards parents.

### “Knowledge is power” - Interest in genetic testing

Quotes to illustrate participants’ interest in genetic testing for schizophrenia risk are shown in Table 3. The majority of participants were in favour of genetic testing for schizophrenia, and their perceptions overwhelmingly related to their own experiences. Most participants who were in favour of genetic testing said they would have a genetic test regardless of the degree of certainty generated and maintained the view that “knowledge is power”. A small number of these participants, however, perceived genetic testing to be less useful if the test result was uncertain. A few participants were not interested in genetic testing. The most frequently mentioned reason for lack of interest was that there was limited benefit if the person was already diagnosed with schizophrenia. Other reasons for lack of interest were that genetic testing was not an exact science; genetic testing was less useful if the person already knew that they were at increased risk because of their family history; knowledge of genetics reduced hope; and that there were too many external factors to consider.

**Table 3:** “Knowledge is power” - Interest in genetic testing.

Themes	Quotations
Interest in genetic testing depending on the degree of certainty	[Interviewer: would you be interested if the test gives you a probability like a 30% to 70% chance. Not exact, but just a percentage]
	*Still interested if not 100% certain (11)*
	Yes, it would not change my mind, I would like to know. (Timothy, unaffected)
	Yes, I’d still be interested. (Andrea, affected)
	*Less useful if not certain (3)*
	Oh no, not 30%. I would not bother. If it was higher than 50%, I would. (Jennifer, unaffected)
	I think it is chance then that would not be very helpful. I think it needs a yes or no. (Eleanor, affected)
	If it [prenatal testing] was 100% they were going to get it, I’d think about it, but if it was only 50% or 60% or 30% or something like that, I still would have had kids. (Vince, affected)
Not interested in genetic testing	*Not an exact science (2)*
	I’m not really that keen on too much of this genetic stuff. Science can make mistakes. They can test you for genes and then put it into the wrong bottle or something and make a mistake. I don’t think it’s the answer to everything. (Tom, affected)
	*Already diagnosed so limited benefit (5)*
	If you have got schizophrenia, it really doesn’t change anything, because whether the genetic test comes back yes or no, you are still suffering with the illness, you know. (Justine, affected)
	*Already know at increased risk because of family history so genetic testing less useful (1)*
	You know it’s in the family- if they came up and said, “Yes, you have got the gene,” I mean it’s not really going to surprise you much, is it, because you really- you already know. (Justine, affected)
	*Knowledge of genetics reduces hope (3)*
	Oh, look I’d be devastated because it takes all the hope out of it all, because at least in my experience. (Tom, affected)
	*Too many other external factors to consider (1)*
	Genetic testing may – there’s so many factors. The genetic test is only one of them. It’s the environment, the stability, beliefs in God, there’s so many, there’s so many and even ones I haven’t come across yet. (Tom, affected)

### Genetic testing provides opportunities for early intervention and avoiding triggers - Perceived benefits and risks of genetic testing

Quotes to illustrate participants’ perceived benefits and risks of genetic testing for schizophrenia risk are shown in Table 4. A wide range of benefits of genetic testing for schizophrenia risk were identified. Many participants took the view that genetic testing provided opportunities for early intervention and avoiding triggers. Other benefits of genetic testing described included that genetic testing may allow for personalised medication; the individual wants to see if it is passed down to children/to make childbearing decisions; genetic testing clarifies risk to other family members; it would help clarify diagnosis; and an increased-risk genetic test result would promote personal acceptance of the condition and adherence to medication. Most saw early intervention or prevention in young people as a key potential benefit. Several opportunities for intervention in at-risk youth were mentioned, including education about warning signs of schizophrenia.

**Table 4:** Genetic testing provides opportunities for early intervention and avoiding triggers - Perceived benefits and risks of genetic testing.

Themes	Quotations
Benefits of genetic testing – Knowledge is power	*Clarifies risk to other family members (3)*
	If it is in the family and if it might tell if anyone else in the family may develop it. (Eleanor, affected)
	*Helps clarify diagnosis (1)*
	I wish there was a test so that he [psychiatrist] would just stop labelling me with so many different [diagnoses]. (Eleanor, affected)
	*Increased-risk genetic test result would increase acceptance and adherence to medication (2)*
	If I had a genetic test that said look you are very likely to get it, that would have made me a lot more likely to accept the diagnosis and to take medication. (Harry, affected)
	*Knowledge is useful, powerful (8)*
	So I am someone who wants to know the truth despite how unpleasant it might be. (Elisabeth, affected)
	I think knowledge is power, so the more information we get, the better we can deal with it. (Vince, affected)
	Well, if you think … have anything… that’s what education is. (Ellen, unaffected)
	*May allow for personalised medication (4)*
	It maybe could lead to some for treatment or something like that. (Michael, unaffected)
	If you have the diagnosis, it is in your genetics, or maybe they can develop a tablet which you can take that stops it developing or something. (Robyn, affected)
	*Provides opportunity for early intervention (6)*
	Be aware of what the warning signs are and be aware that it could be schizophrenia and if it was schizophrenia, they could act on it earlier and hopefully if they act on it early enough, they might get it before it gets too bad. (Vince, affected)
	Possibly he would do some prevention or something like that. (Michael, unaffected)
	*Provides opportunity to avoid triggers if positive (5)*
	Once people know that they may carry the DNA regions, it might be advised by a doctor to maybe lifestyle choices, like do not smoke cannabis, do not take amphetamines you know, maybe try to have a less stressful, mindfulness kind of life. (Will affected)
	Maybe it can definitely help people to keep away from triggers such as stress and drugs. (Timothy, unaffected)
	*Wants to see if it is passed down to kids/to make childbearing decisions (4)*
	Well, I guess if you have both parents tested, than you would know if it is more likely or less likely your own child have it, would get it. (Eleanor, affected)
	I’d have some assurance I’m not going to pass the gene… (Timothy, unaffected)
Risks of genetic testing – The insurance industry would go to town on you	*Insurance and employment discrimination (4)*
	Well certainly the insurance industry would go to town on you. (Tom, affected)
	You have got the possibility of employers being prejudiced against people who have got a high risk. (Ian, affected)
	*Psychological distress if not yet diagnosed and at increased risk (4)*
	Before you get schizophrenia, if you know there’s a certain tendency to get it, it may affect people, give them some kind of like depressions or something like that. (Michael, unaffected)
	But, yeah, definitely it could stress them out and that could even bring on the onset of it and could make them worry a lot and that sort of thing. (Harry, affected)

Only two risks of genetic testing were mentioned by several participants, namely insurance and employment discrimination. Several participants believed evidence of genetic predisposition to schizophrenia may affect their ability to obtain health insurance. Some participants were concerned that they may face discrimination from employers or not be allowed to pursue education. Another concern mentioned was the potential for psychological distress in asymptomatic individuals learning of a predictive result. Some worried this may even act as a trigger in someone who has not yet developed symptoms. This was considered particularly concerning in the context of testing children or adolescents. Some believed children should not be tested at all and should make an informed decision when they were older.

### “Playing God” - Attitudes to reproductive genetic testing

Quotes to illustrate participants’ attitudes to hypothetical reproductive genetic testing are shown in Supplementary File 2. Many participants said knowledge of their or their partner’s personal genetic risk would aid them in making reproductive decisions and perceived this as a benefit.

When asked about a hypothetical pre-implantation genetic test, i.e., being able to select an embryo that has a lower risk of developing schizophrenia, many participants were in favour, and this was generally perceived as being more acceptable and less problematic than prenatal genetic testing. A few participants viewed pre-implantation testing as “playing God” but were not necessarily against it. Many believed informed decision-making in the context of prenatal and preimplantation genetic testing was essential.

Participants were also asked about a hypothetical prenatal test done in the early weeks of pregnancy. Almost all were in favour of such a test being available, and most said they would choose to have it done; however, no participant said they would definitely proceed with a termination if they received a high-risk result. Many likened this hypothetical test to existing prenatal screening tests. Most said it is something they would need to discuss further with their partner and some participants were against termination or selectivity and viewed it as “unnatural” or “not right”.

### “It snowballs” - Understanding of genetics

When asked about their understanding of the genetic aspect of schizophrenia, several participants believed that there were multiple genes involved, and reasons mentioned were that there were varying degrees of mental illness, and that mental illness tended to get worse over time. One participant said that “one triggers another… it snowballs”. Others said they “didn’t know” or had “no idea” about the genetics of schizophrenia. Two participants held simplistic views of genetics in that they attributed schizophrenia risk to single genes. Almost the entire sample expressed an interest in receiving more information about the genetics of schizophrenia.

## Discussion

Most participants endorsed a model of causation for schizophrenia consisting of a combination of environmental and genetic factors, consistent with current scientific consensus [27]. A genetic explanation for schizophrenia was discussed in the context of reduced self-blame and alleviation of guilt, perhaps because a genetic model of causation removes the locus of control from the individual [28]. This would be consistent with the framework of *attribution theory*, which suggests reduced perceptions of controllability of behaviours increase sympathy and reduce blame [29].

Some participants believed a biogenetic explanation for schizophrenia would also reduce stigma from the public and increase acceptance, again due to a shift in the locus of control from the individual, consistent with attribution theory [29] and a recent systematic review [30]. Other participants believed causal attributions would not reduce stigma. While evidence suggests a genetic explanation does in fact reduce blame placed on individuals, biogenetic explanations also increase the public’s desire for social distancing from people with schizophrenia and do not decrease perceptions of ‘unpredictability’ and ‘dangerousness’ [31, 32]. These contradictory findings can be accounted for by an essentialist view of genetics. *Genetic essentialism* postulates that cognitive biases associated with essentialist thinking are elicited when people are presented with a genetic model of causation [33]. Biogenetic explanations for schizophrenia are seen as deterministic or immutable, and hence those affected are perceived as homogeneous and inherently different from others, reinforcing and legitimising stereotypes about mental illness [34]. Promoting biogenetic models of causation thus may not be useful to decrease stigma, even if blame placed on individuals is reduced.

Participants showed considerable interest in genetic testing for schizophrenia risk, consistent with findings from other studies that assessed interest in genetic testing for schizophrenia risk in young people at high clinical risk [35] and unaffected relatives of people with schizophrenia [16, 17]. The presentation of various risk frames only impacted a small minority of participants’ decisions to undergo hypothetical genetic testing, in contrast to other (quantitative) studies, which found that interest was correlated with the positive predictive value of the genetic test [35, 36].

Participants named many benefits they hoped would accompany genetic testing, including that it may allow for personalized medication, provide opportunities for treatment and early intervention, motivation to avoid triggers and help clarify diagnosis, underscoring results from similar studies [17, 35]. In particular, avoiding cannabis use in those identified as being at high risk could have a significant impact on one’s mental health [37], and a recent experimental study shows that young adults would be motivated to abstain from marijuana use if they learned their genetic makeup meant that marijuana use would increase their schizophrenia risk [38].

Risks of genetic testing mentioned by participants related to potential insurance and employment discrimination and psychological distress, particularly in individuals who have not been diagnosed but have been found at high risk. This aligns with other studies in young people at increased clinical risk [14] and unaffected relatives from high-risk families with schizophrenia [17], which found that informing an individual that they were at increased risk could lead to anxiety and fear, might generate a self-fulfilling prophecy and could increase discrimination by others [14, 17].

In contrast, hypothetical genetic testing of pre-symptomatic individuals, particularly young people, was perceived as a key potential benefit, perhaps associated with notions of relatively poor treatability and the benefit of early intervention. Testing of children or adolescents is ethically complex and must take into consideration that decisions will usually be made by the minor’s parents. Potential benefits, such as early intervention with medication or cognitive therapy, must be weighed with potential harms, such as the negative psychological implications of a high-risk status [39].

Almost all participants were interested in a hypothetical prenatal test done in the early weeks of pregnancy, but no participant said they would definitely proceed with a termination based on genetic test results. A previous study involving unaffected people from families with multiple affected family members with schizophrenia [17] similarly showed strong interest in prenatal testing, with 56% reporting that they would want prenatal testing if available. If prenatal testing for schizophrenia risk were to become available, individuals affected with schizophrenia and unaffected people at increased familial risk will need genetic counselling to ensure they understand the predictive accuracy of the test with regard to the likelihood of a child developing schizophrenia. The impact of tests showing substantial risk for schizophrenia on parent-child relationships, given that many pregnant couples may decide against termination, is just one of the questions that will need to be addressed before wide-scale adoption of prenatal testing.

In this study we found that several participants correctly believed that multiple genes contributed to schizophrenia risk. These sound beliefs may be because polygenic risk aligns nicely with lay conceptions of inheritance, which are based on the concept of bilateral kinship, i.e. that offspring receive about half of their genetic material from each parent [40–42]. By contrast, two participants held simplistic views of genetics, in that they attributed schizophrenia risk to single genes. Attributing schizophrenia to single genes may lead to an ‘all or nothing’ of disease aetiology and development and contribute to fatalistic views about schizophrenia. Such views are consistent with binary thinking about genetics [43, 44]. Such misconceptions highlight the need for education about genetics to prevent misconceptions about the condition. Indeed, almost all participants, regardless of their attributions of schizophrenia risk to multiple or single genes, expressed an interest in receiving more information about the genetics of schizophrenia. Delivering such information online and targeting education to individuals with a family history as those at highest risk has been trialled successfully in the bipolar disorder setting [45], and this strategy may also be ideally suited to convey education about genetic aspects of schizophrenia.

This study has several limitations. Participants were highly educated compared to an affected population, and thus the beliefs of people with lower levels of education may be underrepresented. Participants were also quite old, and this could have led to specific generational points of view related to prenatal testing and pregnancy termination. Some unaffected individuals were first-degree relatives of the affected responders. Therefore, they are likely to share similar sociodemographic and cultural background characteristics, thus further narrowing the panel of answers. More than half of the participants were recruited from the ASRB participant register, and as such these participants have been involved in previous research that may influence their familiarity with genetic contributions to schizophrenia and hence their views expressed in the interviews. Additionally, participants with a diagnosis of schizophrenia all had insight into their illness and were sufficiently high functioning to participate in the recruitment process and engage with the interview questions. This high degree of functioning may suggest they are not representative of the broader population of people with a diagnosis of schizophrenia. Due to the qualitative nature of this study, the results cannot be generalized to a larger population, and causal relationships cannot be established, but the findings are transferable to similar settings. Further research may involve quantification of these findings through larger quantitative studies. Discussion of genetic testing in this study was based on a hypothetical genetic test, meaning participant views may not be directly applicable to a genetic test that may become available in the future.

In conclusion, this study reveals a strong interest in both diagnostic and predictive genetic testing by people with schizophrenia and those who are genetic relatives, as well as a strong endorsement of a genetic model as a contributor to causation, nuanced within the context of gene-environment interactions. Genetic testing may provide a degree of optimism, particularly when it comes to alleviating self-blame and making reproductive decisions.

### Data sharing

Data are available from the corresponding author on reasonable request.

## Supplementary information

Supplementary File 1

Supplementary File 2
