# Supplementary material for: Perceptions of causal attribution and attitudes to genetic testing among people with schizophrenia and their first-degree relatives
Source: Eur J Hum Genet. 2022 May 16;30(10):1147–54. doi: 10.1038/s41431-022-01116-8 (PMC9553941; doi:10.1038/s41431-022-01116-8)
Supplement: Supplementary file 1 [file 41431_2022_1116_MOESM1_ESM.doc]

**Supplementary File 1: Interview schedule**

(1) People have different sorts of explanations for schizophrenia.

1. What did you believe causes schizophrenia before you (or your relative) became involvedwith One Door Mental Health?
2. To what extent do you believe inheritance is responsible for schizophrenia in your family?
3. Since you (or your relative) became involved with One Door Mental Health, in which way have your explanations for schizophrenia changed?
4. How does having a genetic explanation for schizophrenia affect the way a person with schizophrenia feels about the illness (e.g. blame, stigma)?
5. How does having a genetic explanation for schizophrenia affect the way other people feel about a person with schizophrenia (e.g. blame, stigma)?

(2) Schizophrenia can affect families in different ways. What has been the impact in your family of having several members of the family with schizophrenia?

(3) Now we would like to talk a little bit about genes for schizophrenia.

1. Do you feel that there is a particular gene that confers risk to schizophrenia?

(ii) Alternatively, do you consider that there are multiple genes that confer increased risk for schizophrenia?

(4) If we were to say that there is a genetic test for schizophrenia, would you be interested in having genetic testing?

(i) Would you be interested, if the genetic test gave you a clear yes/no answer?

(ii) Would you be interested if the test would only give you a probability of how likely it is that you (or another family member) would develop schizophrenia, eg. 30% or 70% change?

(iii) What do you feel are the benefits of genetic testing for schizophrenia?

(iv) What do you feel are the risks of genetic testing for schizophrenia?

(v) What are your feelings about testing children and/or adolescents?

1. Has your diagnosis of schizophrenia (or: the diagnosis of schizophrenia in your relative) impacted on your decision about whether or not to have children?

If we were to say that there is a genetic test for schizophrenia, would you be interested or would you have been interested in genetic testing during the early weeks of pregnancy or testing of embryos conceived through IVF to detect gene variations associated with schizophrenia?

1. It would be helpful to us to know what your preferences are with regard to receiving information regarding genetic risk for schizophrenia.
2. How interested would you be in receiving more information about genetic risk for schizophrenia?
3. How would you like to receive information about genetic risk for schizophrenia? (Prompts: Information leaflet, question prompt sheet, information video, consultation with expert, talks provided by experts, Internet etc)
4. What topics would you like to have covered? (Prompts: my risk for schizophrenia and risk to offspring, early diagnosis, prevention strategies for diagnosis, how to cope with family vulnerability etc)

# Closure

- Debriefing-
  - - How does participant feel after participation in the interview?
    - Inform participants that if participation has raised any needs for information that they should write down their questions and speak to their doctor.
    - Inform participant that if participation has raised any negative emotions that they can speak further with the research staff or research staff can provide a contact number for a support service.
