# Supplementary material for: Perceptions of causal attribution and attitudes to genetic testing among people with schizophrenia and their first-degree relatives
Source: Eur J Hum Genet. 2022 May 16;30(10):1147–54. doi: 10.1038/s41431-022-01116-8 (PMC9553941; doi:10.1038/s41431-022-01116-8)
Supplement: Supplementary file 2 [file 41431_2022_1116_MOESM2_ESM.docx]

**Supplementary File 2:** **“Playing God” - Attitudes to reproductive genetic testing**

| Themes | Quotations |
| --- | --- |
| Attitudes to prenatal testing | *Interested in prenatal testing (10)*  I mean people doing it with Down syndrome anyway, people are doing it with other genetic diseases, yeah, why not? Why not? I mean if they’re gonna get a test for Down’s syndrome or, I don’t know, some of the other dreadful things … might as well test the [schizophrenia] DNA regions as well’ (Will, affected).  Yes, knowing what I know now yes I certainly would have [prenatal testing], yes. (Annabel, unaffected)  *Not interested in prenatal testing (5)*  Oh well it’s too late then no I don’t think I’d bother then. (Jennifer, unaffected)  It would be up the wife. (Tom, affected)  *Unsure (1)*  I wouldn’t want to get pregnant and then have to - that makes me think about people if they find out that their child is going to have schizophrenia will terminate the pregnancy. That’s what I think – I don’ t think I could do that. (Eleanor, affected) |
| Attitudes to preimplantation genetic testing | *Interested in preimplantation genetic testing (7)*  You’re going to be looking after this kid for the rest of your life, so, hey, you know, might as well go with the best embryo, huh?. (Will, affected)  Yes it should be available, but it should be made very clear to the person receiving the IVF, you know, they should be fully informed about these things, about risks … they should have a clear understanding, talking to a doctor, a medical professional about it, I mean I think it’s vital’ (Anna, affected). (Anna, affected)  *Not interested in preimplantation genetic testing (3)*  I don’t think so. [No] No. If I look at my sister, life has been up and down, pretty much like anybody's. [Of course] She has still lived a full life, an eventful life, so I don’t see it as a debilitating thing. So no, I wouldn’t think so. (Oliver, unaffected)  In other cases I’ve heard in the media that this sort of thing is like playing God. It’s like selecting Superman or something, you know, the German Superman idea. (Elizabeth, affected)  *Unsure (1)*  Sorry I’m not sure really. (Timothy, unaffected) |
